# Loneliness and Social Isolation Interventions: A Public Health Analysis

**DOI:** 10.1093/geroni/igaf122.196

**Published:** 2025-12-31

**Authors:** Christina Victor

**Affiliations:** Brunel University London, Guildford, England, United Kingdom

## Abstract

The 1948 World Health Organisation (WHO) definition of health identifies three distinct domains of health: physical, mental and social. Until the second decade of the 21st century the concept of social health was largely neglected. This position was transformed in November 2023 when WHO declared that ‘loneliness as a ‘global public health concern’ and created the Commission on Social Connection. These developments are the culmination of a body of work that has drawn attention to the association between loneliness/social isolation and a range of health and health service use outcomes. A public health approach to loneliness involves primary prevention, proactively preventing loneliness; secondary prevention, identifying and intervening with those at ‘high risk’ of loneliness whilst tertiary prevention focuses upon minimising the consequences of established loneliness. Using a public health lens, we undertook a critical evaluation of 50 loneliness interventions for community dwelling older adults identified through existing systematic reviews from 2010 onwards. Twenty interventions focused upon secondary prevention, ten on primary prevention and was unknown/not reported for the remainder. The duration of interventions was predominantly short term with 55 being completed in under 6 months. Evidence for effectiveness is limited as sample sizes are small with 16 studies having 100+ participants. Approximately half the studies reported a post intervention statistically significant decrease in loneliness scores. This does not easily translate into how score changes enhance the quality-of-life older adults. We suggest that future research should look at identifying score changes which are meaningful to individuals alongside statistical tests.

